# Concomitant immunity in persistent *Leishmania* infections: could it represent an evolutionary balance and a vaccine target?

**DOI:** 10.1186/s13071-025-07188-x

**Published:** 2025-12-08

**Authors:** Francesca Divenuto, Simona Gigliotti, Grazia Pavia, Fabrizio Vitale, Sofia Cortes, Carla Maia, Nadia Marascio, Angela Quirino, Giovanni Matera

**Affiliations:** 1 Clinical Microbiology Unit, Department of Health Sciences, “Magna Græcia” University of Catanzaro, 88100 Catanzaro, Italy; 2 National Reference Center for Leishmaniasis (C.Re.Na.L.), Istituto Zooprofilattico Sperimentale Della Sicilia, 90129 Palermo, Italy; 3 Unidade de Parasitologia Médica, Global Health and Tropical Medicine (GHTM), LA-REAL, Instituto de Higiene E Medicina Tropical (IHMT), Universidade NOVA de Lisboa, Lisbon, Portugal

**Keywords:** Concomitant immunity, T cells, Parasite persistence, Exhaustion, Immunodepression, Reinfection, Vaccines

## Abstract

Concomitant immunity (CI) can be viewed as an example of coevolution between the microorganisms and their long-lived hosts. Such an ecological trade-off may be advantageous to both the microbe and the host, as it allows protozoa and helminths to maintain their genetic features while providing the host, particularly mammals, with long-standing protection against reinfection by the same microbe. In *Leishmania* infection, CI is the mechanism whereby parasites remain at low-level infection in the host, which develops a strong immune reaction that protects against reinfection. Mechanistically, several CD4^+^ T cell populations seem to be involved in such fine immune responses. While immunity against *Leishmania*, *Plasmodium*, *Taenia*, *Schistosoma*, and *Echinococcus* is well known, the mechanisms of CI involving these pathogens have been poorly studied. Finally, the phenomenon of CI should be carefully assessed in the design of novel vaccine preparations.

**Graphical Abstract:**

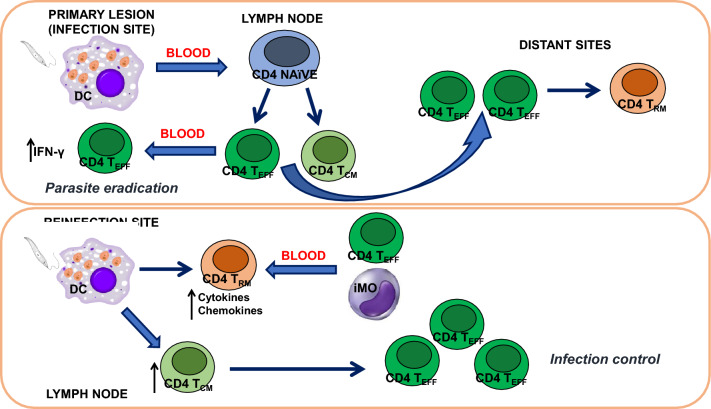

## Background

Asymptomatic and persistent infections increase the likelihood of transmission between long-lived hosts such as humans, thus representing an advantage for pathogens. This symbiosis between pathogen and host can be facilitated by “concomitant immunity” (CI), in which resistance to reinfection is promoted by the persistence of the same species/strain that caused the initial infection (homologous CI) or different species/strains (heterologous CI), thus providing benefits not only for the pathogen but also for the host [1, 2]. The term “concomitant immunity” was introduced in the 1980s in the field of cancer immunology [3].

**Historical example of concomitant immunity**: A historically plausible example of CI could explain the lack of leishmaniasis-like illness among Alexander the Great’s soldiers. King Alexander of Macedonia expanded his kingdom to include some of India’s territories. Neither Alexander nor his soldiers apparently suffered from leishmaniasis, probably because they came from Greece, a country endemic to leishmaniasis. Most probably, contact of such people with *Leishmania* during their infancy established a CI lasting for most of their lives, which would protect Alexander and his soldiers when they likely came in contact with *Leishmania donovani* in Indian territories [4].

CI is considered the “gold standard” against subsequent infections, even if it does not confer sterile immunity following infection [5]. *Leishmania* sp., as well as *Mycobacterium tuberculosis* and *Toxoplasma gondii*, can persist indefinitely in the host in low numbers without causing manifest diseases [6].

These “persistent” infections are different from “chronic” infections, which are often symptomatic and characterized by a much higher number of pathogens [6]. A key role in resistance to reinfection is played by a residual pool of memory T cells. The characterization of memory T cells and the study of the mechanisms involved in their generation and maintenance are of particular importance for vaccine development [5].

Three major subsets of memory T cells have been identified: tissue-resident memory T (T_RM_) cells, effector memory T (T_EM_) cells and central memory T (T_CM_) cells [7]. T_RM_ cells reside in non-circulating tissues such as the skin, lung, liver and intestinal tract, where they act as sentinels, providing immediate protection against pathogens. T_EM_ cells circulate between the blood and lymphoid and non-lymphoid tissues. These cells can rapidly differentiate into effector cells in the presence of the specific antigen, and they quickly identify and eliminate recognized pathogens. T_CM_ cells reside mainly in the lymph nodes and spleen. They have the capacity for self-renewal and can differentiate into effector T cells [8].

In CI, the “gold standard” of protective immunity against phagosomal infections is CD4-mediated protection [9]. After resolution of infection with wild-type *Leishmania major* parasites, the skin of cured mice harbours CD4^+^ T_RM_ cells that are essential for optimal immunity against *Leishmania* reinfection [10]. The same protection is achieved in the practice of leishmanization. This method provides protection against CL by intradermally inoculating a live, attenuated form of the *L. major* parasite to create a self-healing skin lesion, which induces a protective immune response against future natural infections. Despite the establishment of a protective immune response capable of mitigating the disease, secondary infections caused by *L. major* are still able to establish themselves effectively. However, they have lower clinical manifestations in previously infected hosts.

Mandell and Beverley [11] proposed a model of CI in which persistent parasites serve as a natural vaccine that continuously renews itself. Persistent infection parasites (PIPs) have two populations: one replicates rapidly as in acute infection, while the other is quiescent or replicates very slowly. The progeny of replicating parasites have two potential fates: some parasites repopulate the pool of quiescent or replicating parasites, while others are destroyed, maintaining low parasite populations and providing antigen for continued immune stimulation throughout the host’s life [11]. Although much is known about the nature of protective immune responses against *Leishmania* sp., many aspects remain unclear. Therefore, this narrative review considers the phenomenon of immunity to *Leishmania* reinfection in the animal and human hosts.

## Box 1. Definitions and distinctions in *Leishmania* Infections

**Table:** 

Concomitant immunity (CI)	versus	Premunition
A state of partial protection against reinfection that persists as long as the host retains a small residual population of parasites from the primary infection. This protective effect depends on the persistence of these parasites and on the activity of memory and effector T cells, particularly IFN-γ-producing Th1 cells [1, 6]		Historically, this term (which applies to both malaria and leishmaniasis) describes the protection provided by an ongoing infection against superinfection. This concept includes both symptomatic and asymptomatic states and does not necessarily involve a defined immunological mechanism [12, 13]
Homologous CI	versus	Heterologous CI
CI that protects against reinfection with the same *Leishmania* species/strain as the primary infection [1, 2, 6]		CI that extends, partially or fully, to reinfection with different *Leishmania* species/strains [1, 2, 6]
Persistent infection	versus	Chronic infection
A low-level infection characterized by a very limited parasite burden (∼10^3^ parasites), usually asymptomatic, under immune control, and able to reactivate a full infection during immunosuppression conditions [6]		It is an infection with higher parasite loads, often accompanied by clinical symptoms and tissue pathology, reflecting ongoing inflammation and less effective immune containment [14]

## Parasite persistence in cutaneous and visceral leishmaniasis

The complex life cycle of *Leishmania* parasites involves vertebrate and invertebrate hosts and two developmental stages: flagellated promastigotes are found in the female sand fly’s gut, while they transform into amastigotes within mammalian host cells [15]. Leishmaniasis is caused by several *Leishmania* species, which are responsible for the different clinical manifestations of the disease. The characteristics of the parasite, the biology of the vector, and the host immune response are decisive in the outcome of the disease. There are three main clinical forms of human leishmaniasis: visceral, cutaneous and mucocutaneous leishmaniasis. *Leishmania donovani* and *L. infantum* cause visceral leishmaniasis (VL) or kala-azar, the most severe form of disease characterized by fever, weight loss and enlarged liver and spleen. Cutaneous leishmaniasis (CL) in the Old World is mainly caused by *L. major*, *L. tropica* and *L. aethiopica*, while in the New World it is mainly caused by *L. amazonensis*, *L. mexicana*, *L. braziliensis* and *L. guyanensis*. CL presents skin ulcers that usually heal spontaneously but can also lead to scarring and disfigurement. Mucocutaneous leishmaniasis (MCL), also known as “Espundia”, is caused by species such as *L. braziliensis*, *L. guyanensis* and *L. panamensis*, and it is characterized by disfigurement of the oral and nasopharyngeal mucosa [16].

**Fig. 1:**
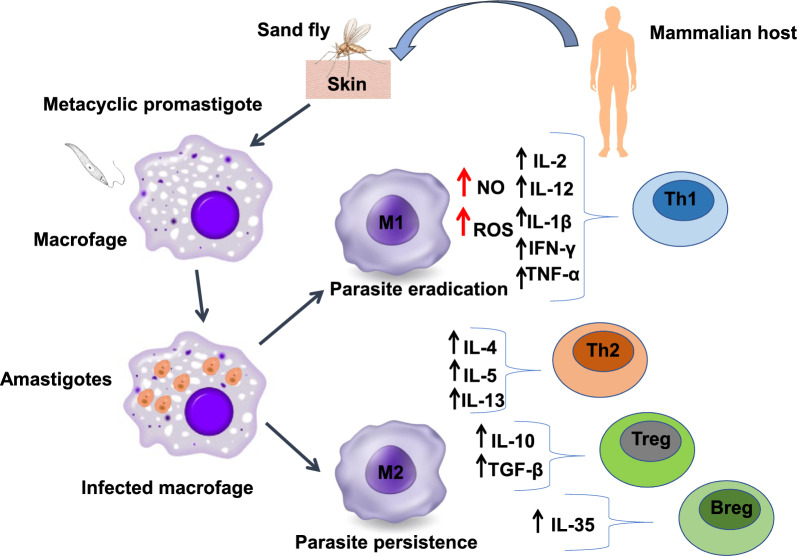
*Leishmania* infection can promote the development of M1 or M2 macrophages, associated with either Th1 or Th2 immune response, respectively. The polarization of macrophages into pro-inflammatory (M1) or anti-inflammatory (M2) phenotypes drives the immune response in leishmaniasis. The M1 phenotype is associated with parasite eradication while the M2 phenotype is associated with parasite persistence. Pro-inflammatory cytokines (IL-1β, IL-2, IL-12, IFN-γ, TNF-α) induce parasite killing. Anti-inflammatory cytokines (IL-4, IL5, IL-10, IL-13, IL-35, TGF-β,) favour parasite survival and disease progression

*Leishmania* parasites adopt strategies to evade the host’s immune system, surviving in macrophages and maintaining the infection. Parasite eradication relies mainly on macrophage-derived reactive oxygen species (ROS) and nitric oxide (NO), induced by Th1-mediated activation of M1 macrophages that produce pro-inflammatory cytokines such as interferon gamma (IFN-γ), tumor necrosis factor alpha (TNF-α), interleukin (IL)-1β, IL-2 and IL-12 that lead to macrophage activation and eliminate amastigotes [17–19]. Some parasite cell surface molecules, such as the metalloprotease gp63, are involved in several escape mechanisms, altering host macrophage signalling [18, 19]. Studies in the mouse model showed that not only IL-1β production but also inflammasome activation are involved in the control of *Leishmania* infection in vivo. These multi-protein complexes are assembled in the cytoplasm of innate immune cells, where they represent a defence mechanism. However, *Leishmania* parasites evolved mechanisms to evade inflammasome activation, for instance by reducing ROS, which are important in the activation process of the most studied NLRP3 inflammasome [20]. The clinical severity of the disease is associated with polymorphisms of the human IL-1β gene [21, 22]. Resistance to leishmaniasis is linked to the development of a Th1 cellular response, with the production of pro-inflammatory cytokines such as IL-12, IL-1β, IFN-γ, TNF-α and IL-2, leading to macrophage activation profile M1 through the production of NO and ROS and parasite killing. *Leishmania* has evolved different strategies to survive in macrophages: the inhibition of NO and the increased production of immunosuppressive molecules such as IL-10, IL-13 and transforming growth factor beta (TGF-β) (a macrophage activation profile known as M2, associated with the Th2 immune response). Parasite resistance within macrophages after apparent clinical cure or treatment, is related to intense Th2 (IL-4, IL-5, IL-13), regulatory T (T reg IL-10 and TGF-β) and regulatory B (Breg IL-35) responses [15, 17, 23] (Fig. 1). Regulatory lymphocyte subpopulations (Treg and Breg cells) inhibit macrophage and effector T cell activation that are crucial in fighting *Leishmania* infection [21]. The contribution of Treg cells to the establishment of CI against CL may represent a unique process occurring in the skin. It is well documented that leishmanization, the intradermal inoculation of viable parasites from a lesion from a CL-infected individual at a specific skin area of a naïve individual, induces a CL lesion which, after healing, protects the individual from further natural *Leishmania* infections for life [24].

Studies on the complex dialogue between low parasite levels and host immune responses have suggested that CI may develop in patients infected with *L. major* [25]. The persistence of the *Leishmania* parasite has been studied and documented in both human and animal models of infection. However, there are still unclear aspects regarding the host cells responsible for this persistence. It is reported that host cells involved in *Leishmania* infection include macrophages, fibroblasts, neutrophils, dendritic cells (DCs) and Langerhans cells (LCs) [26, 27]. Viable parasites were found in macrophages and DCs of mice lymph nodes recovered from *L. major* infection. Also, fibroblasts harbouring significant numbers of persistent parasites were found in the lymph nodes of mice healed from skin lesions caused by *L. major* [26]. It has been shown that in *L. major* infection, CD4^+^CD25^+^ Treg cells are recruited within the dermis and contribute, together with IL-10, to the inhibition of effector T cells, promoting parasite survival [28]. The presence of Tregs increased levels of the immunosuppressive cytokine IL-10, promoting the persistence of the parasite. Depletion of CD4^+^CD25^+^ Tregs or blockade of IL-10 secretion leads to a sterilizing cure in infected mice [29]. Treg cells play an important role in the persistence of *L. major* in C57BL/6 mice. In particular, IL-10 produced by Treg cells can promote parasite persistence by modulating the activity of antigen-presenting cells (APCs) or by inhibiting parasite killing in infected macrophages [30]. IL-10 and Treg cells are required to develop resistance against *L. major* reinfection [25].

During VL, three organs are essentially involved: liver, bone marrow and spleen. Differences in the microenvironment of splenic and hepatic tissue are related to differences in the ability to generate efficient immune responses and control of the parasite in these target organs. Macrophages are the preferred targets of *Leishmania* parasites, especially *L. donovani* and *L. infantum* [31]. The resolution of the disease is associated with the formation of a granuloma involving increased expression of inducible NO synthase (iNOS) by macrophages, regulated by several Th1 pro-inflammatory cytokines [32]. Both CD4^+^ and CD8^+^ T cells are involved in resistance and healing against *L. donovani* [33], playing an essential role in granuloma formation [34], characterized by the recruitment of monocytes, macrophages, DCs, eosinophils and neutrophils [32]. The mechanisms by which *L. infantum* and *L. donovani* persist in the host include alteration of the immune response (interference with macrophage activation and signalling pathways) and evasion of programmed cell death (apoptosis) [35].

In addition, in silico (computational) approaches are available to identify pathogen–host interactions and factors promoting parasite spread and disease progression [21]. Finally, both hematopoietic and non-hematopoietic cells can harbour *Leishmania* during the acute, chronic or persistent infection [36].

The production of IL-10 and the inhibition of IL-17 secretion (related to the resolution of infection) by CD4^+^ T cells in humans are stimulated by IL-27. IL-27 is composed of the subunits p28 and Epstein–Barr virus (EBV)-induced protein 3 (EBI3) [37]. High circulating levels of IL-27 and elevated expression of IL-27p28 and EBI3 transcripts were found in patients with VL. IL-17^−/−^ mice infected with *L. infantum* showed increased proliferation of Treg cells and IL-10 with parasite persistence [21]. IL-10 plays a central role in the immune response during human VL, promoting disease chronicity by suppressing host immunity [38]. In the mouse model of *L. infantum* infection, both CD4^+^CD25^+^Foxp3^+^ and CD4^+^CD25^−^Foxp3^−^ (Tr1) Treg populations were identified as sources of IL-10. Similarly, in patients with *L. donovani* infection, these same T cell subsets were shown to produce IL-10 and TGF-β, thereby contributing to the pathogenesis of human VL [26].

Studies have shown a greater permissiveness of monocytes to parasite proliferation than DCs or macrophages in infected tissues. *Leishmania donovani* infection can be characterized by an excessive accumulation of Ly6C^+^ monocytes in bone marrow and spleen, providing a niche for parasite survival and proliferation [39].

## Concomitant immunity and “exhaustion” of T cells in *Leishmania* sp. infection

CD4^+^ T cells play a crucial role in the generation of concomitant anti-parasitic immunity in many parasitic infections, such as those caused by *Leishmania*, *Trypanosoma*, *Toxoplasma* and *Plasmodium* [40]. During the natural host response to *Leishmania* challenge, they mediate the increase in specific immunity to efficiently control parasite infection. After the reduction in parasite number, most of the T helper (Th) cells (short-lived cells) die, and the remaining cells differentiate into memory T cells, promoting parasite control upon re-exposure. However, if the CD4^+^ T cell response fails to control the parasite, a chronic infection process sets in.

During parasite persistence, CD4^+^ Th cells enter a non-functional state known as “exhaustion”, rather than differentiating into classic memory cells. Exhausted T cells have impaired effector functions exhibiting reduced cytotoxicity and cytokine production, allowing *Leishmania* to persist and contributing either to disease progression towards more severe forms or to CI with low parasite loads. Cell exhaustion is reinforced by the increased production of IL-10 and TGF-β by macrophages, which inhibit IL-12 and IFN-γ expression by CD4^+^ T lymphocytes [41, 42].

Increased expression of inhibitory receptors such as programmed death-1 (PD-1) is also a hallmark of T-lymphocyte exhaustion, especially in splenic DCs [42, 43]. Engagement of PD-1 with its ligand PD-L1 suppresses T cell function and activity, while other inhibitory receptors, including lymphocyte activation gene 3 (LAG-3) and T cell immunoglobulin and mucin protein-3 (TIM-3), contribute to this process [44]. IL-35 production by Treg plays a central role in T cell exhaustion by inducing LAG-3, TIM-3 and PD-1 expression on the T cell surface.

**Fig. 2:**
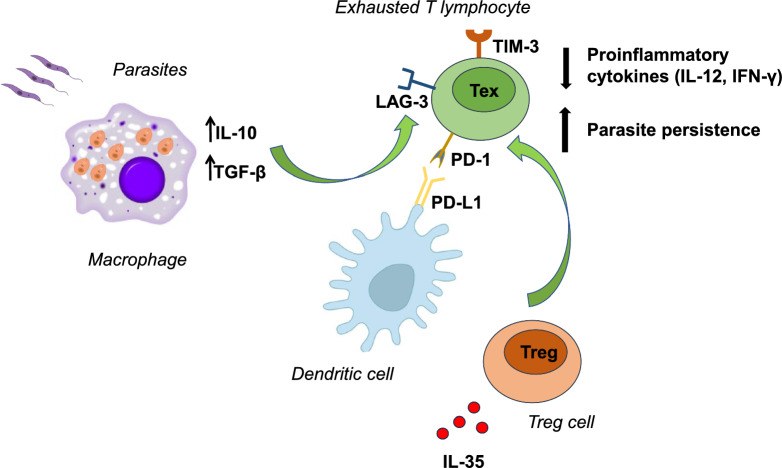
“Exhaustion” of T cells in *Leishmania* sp. infection. IL-10 released by *Leishmania*-infected macrophages causes inhibition of IFN-γ and IL-12 production by CD4^+^ lymphocytes. The PD-1/PD-L1 pathway is involved in the mechanism of T cell depletion, and IL-35 production by Treg plays a central role in T cell exhaustion by inducing the expression of lymphocyte activation gene 3 (LAG-3), T cell immunoglobulin and mucin protein-3 (TIM-3), and programmed death-1 (PD-1) receptors

Exhausted T cells have been described in both VL and CL, but their impact on disease outcome appears to vary depending on the infecting *Leishmania* species [41] (Fig. 2).

Despite clinical resolution, *Leishmania* often persists in the host at low levels, sustaining immune activation and preventing reinfection [45]. This condition, termed CI, is maintained by the antigenic stimulus provided by persistent parasites [46]. It was found that in C57BL/6 mice infected with *L. major*, the persistence of some parasites even after spontaneous clinical recovery stimulates responses based on immune memory and resistance to reinfection. To support this hypothesis, studies indicate that the complete eradication of the parasite results in a loss of protection in infected mice [26].

According to the CI model proposed by Mandell in 2017, PIPs comprise two cell populations: one that replicates similarly to acute-phase parasites in logarithmic growth and a second population that replicates very slowly or perhaps not at all. Importantly, the number of PIPs remains stable over time because parasite replication is balanced by immune-mediated killing. Therefore, replicating parasites can continue actively replicating, enter a state of quiescence/non-replication or be destroyed. Although the mechanisms governing these fates are not fully understood, the continuous replication and subsequent destruction of persistent parasites by the host’s immune system provides an attractive explanation for CI in *Leishmania* resembling a “live” vaccination strategy. The low number of persistent parasites is controlled by the immune system, which allows the host to develop protective immunity against future infections. Killed parasites would provide a good source of antigen to maintain a strong anti-*Leishmania* response at the initial infection site. From the host's point of view, persistent parasites act as a self-stimulating vaccine that continuously renews itself [6].

It can be assumed that the low number of parasites and Treg-mediated suppression may act as a persistent source of antigens necessary to maintain a memory T cell pool [25]. Treg cells contribute to CI through IL-10, TGF-β and IL-35 secretion, inhibitory molecules such as cytotoxic T-lymphocyte antigen 4 (CTLA-4) and LAG-3, and alteration of metabolism through IL-2 deprivation. The balance between parasite persistence ensures long-term memory maintenance while preventing uncontrolled pathology [25].

*Leishmania major* persistent parasites sustain CD4^+^ T cells, which disappear within weeks if transferred into naïve hosts, demonstrating their dependence on antigen persistence [47, 48]. IFN-γ-producing T_RM_ and recruited effector (T_EFF_) cells are crucial for early protection after sand fly *Leishmania* transmission. T_RM_ cells, specialized for barrier tissues, enhance local immunity by recruiting circulating T cells [49, 50].

T_RM_ cells are memory cells evolved to protect epithelial barriers after infection by a pathogen. *Leishmania*-specific CD4^+^T_RM_ cells can increase the recruitment of circulating T cells by boosting immunity [49, 50]. Therefore, these cells may play a primary role in the development of vaccines against *Leishmania* [50].

Scott [46] showed that IFN-γ release by T_RM_ cells during the first hours after infection mediates CCL2-dependent recruitment of inflammatory monocytes (iMOs), leading to increased expression of major histocompatibility complex (MHC) II molecules and production of ROS and NO. This event is associated with an approximately three-fold reduction in parasite load 72 h after infection compared to naïve mice. In lymph nodes, naïve T cells differentiate into T_EFF_ cells after antigenic stimulation: some migrate into uninfected skin tissue, while the rest undergo apoptosis [51]. During a primary infection, promastigotes are deposited in the skin, invading phagocytic cells such as DCs.

**Fig. 3:**
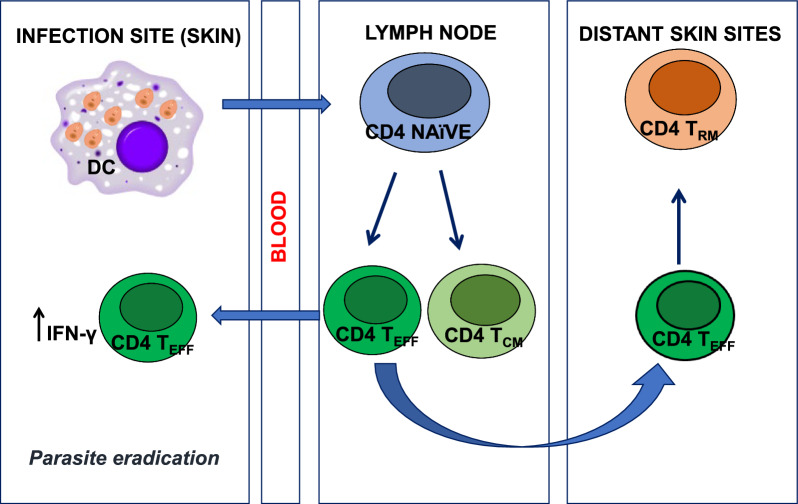
T cell responses after *Leishmania* infection. During a primary infection, promastigotes are taken up by phagocytic cells such as dendritic cells (DCs) at the site of infection (skin). The differentiation of DCs in the lymph node stimulates naïve T cells to differentiate into T_EFF_ or T_CM_ CD4 T cells. Some T_EFF_ cells migrate to the site of infection, leading to eradication of the parasite; the others reach distant skin sites and become T_RM_ cells

DCs migrate from the skin to the lymph node stimulating the proliferation of naïve T cells that differentiate into T_EFF_ or T_CM_ cells. The T_EFF_ cells reach the site of infection and produce IFN-γ, a cytokine that contributes to the eradication of the parasite. Some T_EFF_ cells migrate to distant skin sites, where they become T_RM_ cells (Fig. 3).

**Fig. 4:**
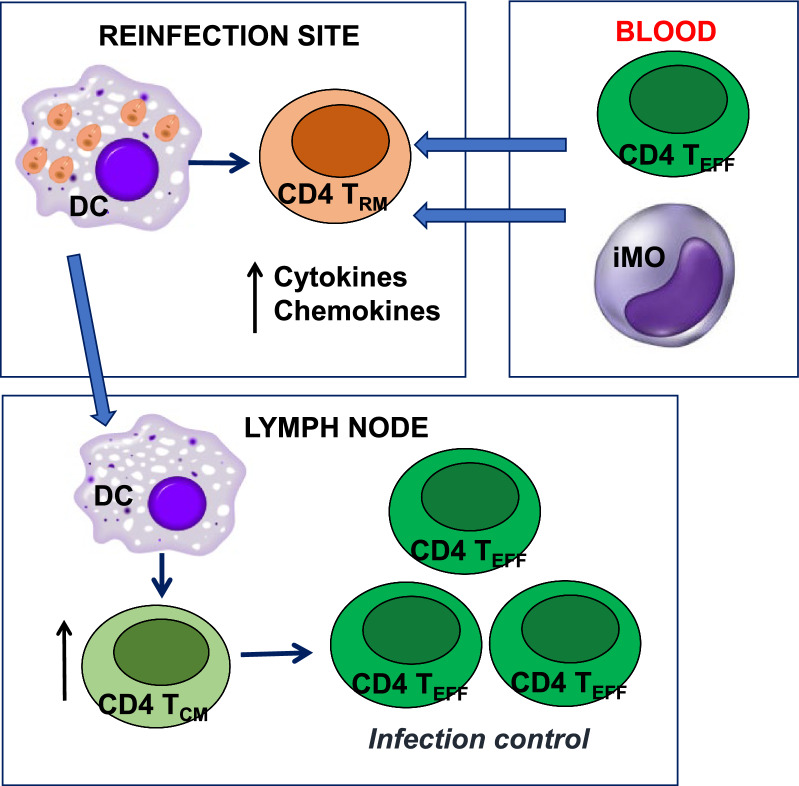
T cell responses following reinfection with* Leishmania* parasites. When mice are reinfected with *Leishmania*, T_RM_ cells produce cytokines and chemokines that recruit T_EFF_ cells and inflammatory monocytes (iMOs). In the draining lymph nodes, DC cells stimulate the proliferation of T_CM_ cells that subsequently differentiate into infection-controlling T_EFF_ cells [10, 51]

After reinfection with *L. major*, T_RM_ cells from healed mice are activated, producing cytokines and chemokines that promote the recruitment of T_EFF_ cells and iMOs. In addition, DCs migrate from the skin to the draining lymph nodes, where the differentiation of T_CM_ cells into T_EFF_ cells is stimulated. The rapid increase in the T_EFF_ cell population enables the control of the secondary infection (Fig. 4) [10, 51].

Understanding this mechanism is crucial to exploiting the role of T_RM_ cells in the development of a successful vaccine [51]. To date, T_EFF_ cells have not yet been characterized in humans, and the mechanism of T_RM_ cell generation is still poorly understood. In areas of high endemicity, CI can help to control the spread of the disease, reducing the incidence of new infections [49].

## Controversies and caveats: absence of detectable parasites in healed *L. major*-induced human CL lesions

Although numerous experimental and clinical studies support the role of low-level parasite persistence in the induction of concomitant anti-parasitic immunity in various *Leishmania* infections [6, 11, 24, 26], some human studies, particularly those involving CL caused by *L. major*, have reported an absence of detectable residual parasites in healed lesions [45]. These observations challenge the universal applicability of the persistence-based model of CI [6, 11]. While the presence of residual parasites in anatomical sites beyond the lesion, such as draining lymph nodes, cannot be definitively excluded, these findings suggest that *L. major*-induced CL may achieve complete parasite clearance at the lesion site, in contrast to other *Leishmania* spp. [45]. This interpretation is consistent with clinical observations in Tunisia reported by Sghaier et al. [45], who found that healed human *L. major* lesions rarely relapse at the original site, suggesting reinfection rather than reactivation from low-level persistent parasites [45]. However, the absence of *Leishmania* genotyping precludes definitive confirmation of this hypothesis. In immunocompromised patients, multiple *Leishmania* species, including viscerotropic species (*L. infantum*, *L. donovani*) and dermotropic species (*L. braziliensis*, *L. infantum*), have been isolated from CL in HIV-positive patients [52–54]. While *L. major*-related CL cases in HIV-positive individuals appear as primary cutaneous infections acquired after HIV infection, the possibility that HIV could trigger reactivation of persistent parasites from previously healed CL lesions cannot be entirely excluded [55–57]. The absence of persistent parasites in clinically healed *L. major*-induced CL [42] raises the possibility that durable protective immunity in this form of CL may be maintained through alternative mechanisms, such as long-lived T_RM_ cells, independent of persistent parasites and CI.

## Concomitant immunity: *Leishmania* versus other parasites

CI is observed across diverse parasitic infections and can explain resistance to reinfection while allowing low-level persistence of the pathogen. In helminth infections, CI often prevents new infections despite ongoing parasite survival. For example, in rodents experimentally infected with *Taenia*, exposure to *Taenia* eggs was found to induce immunity to reinfection [58]. It was also observed that protection from infection in naïve rats occurred even after the injection of serum collected from infected animals [3].

The level of infection that confers immunity and the duration of immunity have not been clarified. It was shown that serum from donors with severe infections is more protective than serum from animals with milder infections. Repeated and frequent re-exposure to the parasite’s eggs can maintain immunity [3]. It has been shown that in CI, the presence of adult worms, cross-reactive antigens between adult and larval stages, and the immune alterations confer resistance to the larval stages of the same organism [59]. On the contrary, eliminating adult worms through spontaneous expulsion or by anthelmintic treatment leads to the establishment of new larvae until a state of equilibrium is achieved [60]. Specific immunoglobulin G (IgG) and IgE antibodies activating eosinophils and macrophages may be involved in resistance against helminths (larval stages). For instance, high levels of anti-*Schistosoma* antibodies are involved in CI in individuals living in endemic areas [59]. In BALB/c mice, prior infection with this trematode reduced worm burden, egg counts and granuloma size upon reinfection. This “self-care phenomenon” typical of CI can be considered similar to vaccination, as live adult worms “vaccinate” the host against a subsequent infection, highlighting the protective effect of the antigens that they release [61]. Although the mechanisms underlying CI remain controversial, long-lived adult worms are coated with host antigens and adopt multiple mechanisms to evade host immunity and simultaneously induce anti-larval immunity, benefiting both host and parasite. The host represents a vital resource for an adult *Schistosoma*. If larval worms successfully establish themselves, they can pose a threat to adult worms by reducing their fitness. Although older worms prevent new infections through CI, they produce few eggs due to reproductive senescence [62].

The CI mechanism has also been observed in infections caused by *Echinococcus* spp., in which the immune system reacts to the initial infection by producing antibodies, activating immune cells and releasing cytokines. *Echinococcus* oncosphere penetration helps to limit new infections, particularly during the larval stages of the parasite: while existing larvae persist, the immune system is often able to limit new infections while maintaining established infections [63].

CI has also been described in other protozoan infections, such as those caused by *Plasmodium*; in human malaria, most individuals are unable to develop true sterilizing immunity, remaining vulnerable to asymptomatic low-charge infections until adulthood. This phenomenon results in tolerance to malaria infection: while it does not protect against the development of symptomatic malaria, it protects against its serious complications. A concomitant state of immunity is acquired in young children who survive early infections [64]. In endemic areas, repeated exposure to the parasite may lead the host’s immune system to control the parasite load, reducing the severity of the disease without eradicating the parasite. The immunity due to previous malaria results in mild symptoms rather than full-blown severe disease [65]. CI is primarily mediated by adaptive immune responses, including the activity of T cells, particularly CD4^+^ T cells, which produce cytokines such as IFN-γ to activate macrophages and control parasite growth and antibodies that target merozoite antigens, preventing red blood cell invasion.

Common to *Plasmodium*, *Leishmania* and *Trypanosoma* infections, persistence of a small number of parasites sustains continuous antigen presentation by APCs, maintaining long-term effector and memory T cell populations providing protection against new infections [66]. In toxoplasmosis, CI is determined by the antigens released by the bradyzoites (encysted forms), which persist for life in host tissues (especially in tissues like brain and muscles) [67, 68] and stimulate an immune response against tachyzoites, the rapidly dividing forms, leading to specific protection against reinfection [69]. In immunocompetent individuals, reinfection is rare, but immunosuppression (e.g., AIDS, chemotherapy) can compromise this immunity, increasing susceptibility to reinfection [68].

To sum up, CI is observed across diverse parasitic infections, where it provides resistance to reinfection while permitting the pathogen to persist at low levels, allowing a controlled, long-term host–parasite relationship.

## Concomitant immunity: advances in *Leishmania* vaccines

The different immune evasion strategies that *Leishmania* deploys to survive within the host create an environment favourable to the persistence of the parasite. This makes the development of effective therapies and vaccines difficult. This is why understanding CI is crucial for vaccine design [70].

An ideal vaccine should mimic a protective environment by stimulating memory T cells and ensuring the development of effector T cells, such as Ly6C^+^ T cells, implicated in parasite control [70]. However, little is known about the role played by T_RM_ cells in vaccine-induced immunity, including that against *Leishmania* [10]. A successful vaccine should mimic the protective immunity observed in naturally infected individuals [71]. To better study the mechanisms in which T_EFF_ and T_RM_ cells are involved, asymptomatic or cured individuals in endemic areas should be studied to improve vaccine design [49]. Live-attenuated strains of *Leishmania* that persist at low levels in the immunized host could be used as vaccines.

The key principles for designing *Leishmania* vaccines based on CI are summarized in Table 1.

**Table 1:** Principles for *Leishmania* vaccine design based on concomitant immunity

Principle	Description	Take-home message
Mimic natural protective immunity	Vaccines should reproduce the immune profile of naturally infected or asymptomatic individuals	Observing immune responses in endemic populations can inform antigen selection and dosing strategies [49, 71]
Stimulate memory T cells	Ensure the induction of long-lived memory T cell populations	Memory T cells sustain immunity and contribute to rapid response upon re-exposure [70]
Promote effector T cells (e.g., Ly6C^+^ T cells)	Support the development of effector populations implicated in parasite clearance	Ly6C^+^ T cells are critical in mediating protection against *Leishmania* [70]
Engage T_RM_ cells	Target T_RM_ cells at potential infection sites, e.g., skin	T_RM_ cells provide localized, rapid immune protection, although their role in vaccine-induced immunity is not fully characterized [10]
Use low-persistence or attenuated live strains	Vaccines may include live-attenuated parasites capable of limited persistence	Low-level persistence can mimic natural CI, stimulating protective T cell responses without causing disease
Study immune mechanisms in endemic populations	Research in asymptomatic or cured individuals can guide vaccine development	Understanding the interplay of T_EFF_ and T_RM_ cells in humans enhances rational vaccine design [49]

## Conclusions

CI represents an adaptive strategy through which the host controls parasitic burden without necessarily achieving complete pathogen clearance. This phenomenon reflects the fine balance between immune protection and parasite persistence, underscoring the complexity of host–parasite interactions and opening new perspectives for research into the effective management and treatment of parasitic diseases. In *L. major* infection, the persistence of parasites in healed lesions appears to contribute to durable protection against reinfection, resembling the principle underlying leishmanization. The T_RM_ cells play a crucial role in this process, orchestrating rapid local immune responses that limit parasite growth. Understanding how CI is established and maintained could inform the development of vaccines and immunotherapies that mimic this natural form of protection. Future studies should focus on clarifying the mechanisms governing T_RM_ cell maintenance, durability and effector functions to optimize long-term immunity against leishmaniasis and other parasitic diseases. These open questions are summarized in Box 2.

## Box 2. Key open questions

**Table:** 

Question	Rationale/implications
Minimal antigenic/parasite load to maintain T_RM_	It remains unclear how much persistent parasite or antigen is required to sustain skin-resident memory CD8^+^ T cells after healing of *L. major* lesions [72]
Durability of T_RM_-mediated protection without live parasites	Understanding whether T_RM_ cells can provide long-term protection in the absence of parasite persistence is critical for vaccine design [72, 73]
Mechanisms of T_RM_ recruitment and effector function	T_RM_ cells recruit inflammatory monocytes that control parasite growth via ROS and NO, but the precise regulatory pathways, including the role of IL-10, remain to be fully elucidated [72]
Species-specific variability of CI	Different *Leishmania* species interact differently with the host immune system, potentially affecting the development and maintenance of CI [72]

In conclusion, CI in leishmaniasis and in other parasitic diseases represents a fascinating aspect of the host–parasite relationship, with significant implications for understanding the pathogenesis of the disease, designing more effective treatments and advancing vaccine development. However, the delicate balance between immunity and parasite persistence remains a significant challenge in both clinical management and research.

## Data Availability

No datasets were generated or analysed during the current study.

## Abbreviations

CI: Concomitant immunity
T_RM_ cells: Tissue-resident memory T cells
T_EM_ cells: Effector memory T cells
T_CM_ cells: Central memory T cells
CL: Cutaneous leishmaniasis
VL: Visceral leishmaniasis
MCL: Mucocutaneous leishmaniasis
NO: Nitric oxide
PD: Programmed death
LAG: Lymphocyte activation gene
ROS: Reactive oxygen species
TIM: T cell immunoglobulin and mucin protein
APCs: Antigen-presenting cells
DCs: Dendritic cells
LCs: Langerhans cells
Treg cells: Regulatory T cells
iNOS: Inducible NO synthase
iMO: Inflammatory monocyte
PIPs: Persistent infection parasites
